# Remarks on the Treatment of Dysentery

**Published:** 1852-10

**Authors:** E. F. Starr

**Affiliations:** Rome, Ga.


From the Southern Medical and Surgical Journal.

*Remarks on the Treatment of Dysentery.* By E. F. STARR, M.D., of Rome, Ga.

This being the season for dysentery, it may be allowable to make a few remarks upon its pathology and treatment.

My only apology for this intrusion is that an astonishing number of deaths from this disease occurs all over the country, seeming to justify a repetition of the opprobious language of Macbeth,

' Throw physic to the dogs—I'll none of it."

I am of opinion that "*physic*" is not so much to blame as *physician.* And as I expect to differ practically, if not theoretically, from many members of the profession, let me suggest that we throw aside preconceived and vague notions and opinions which have been acquired by the process of *taking for granted*, rather than by reflection and observation, and that we come up fairly, without prejudice or favor, to the consideration of this interesting, because common, and fatal disease.

To premise: it will be admitted that dysentery consists in an irritated or inflamed state of the mucous membrane of the lower intestines—usually slight at first, having a tendency to increase to a dangerous extent or to diminish to convalescence, according to circumstances. I need not enumerate its symptoms, as these are sufficiently well known; but the question may be asked, is dysentery a primary disease?—or does it depend upon a depraved state of the secretions?

I must advocate the former position as generally correct, because I see no good reason for believing otherwise. That the predisposing cause of the disease is a morbid impression made upon the nervous system and reflected upon the intestines, I think very probable; but I must protest against the idea that it depends upon a defective secretion of the neighboring organs. I say this much, because the treatment of many practitioners indicates that they are influenced by a different opinion or theory. Yet, we find in some cases of dysentery a deficiency of the bilious secretion in the alvine discharges; but this no more proves the disease in question to be produced by the want of bile, than it does that the deficiency of bile is occasioned by the disease.

Again: there may be an apparent want of this product when there is no real deficiency. In health there is only a sufficient quantity of bile discharged to color properly one evacuation per day. How then is it to be expected that in dysentery a dozen discharges in the same length of time will be equally colored? In dysentery, we have evident mucous inflammation, more or less intense, producing pain, griping, tenderness, mucous and bloody discharges, &c., &c. The indication is plain, to cure the disease by acting upon it directly where it exists. Have we the remedies to answer this indication? We certainly have. Are they to be found in the catalogue of cathartics? When the physician is called to such a case, the bowels have generally been discharging themselves until there are no consistent matters left, therefore the peculiar service of this class of remedies is not needed. If any cathartic possesses the property of curing inflammation of the mucous coat, I do not know it. I know of none upon which we may rely for this. Why then is so much confidence placed in calomel and other purgatives, to the exclusion or partial exclusion of other more efficient and rational remedies? Who does not know that the preparations of mercury in ordinary doses are highly irritating under some circumstances; and that too when such effect is most to be reprehended and guarded against? And will any body believe that an article liable to produce the very effect which we wish to combat is the safest and surest remedy to be used?

Not long since, I happened to hear a man speaking of the prevalence of dysentery in a certain section, and of the fatality which attended it, that scarcely any, of a number of cases which he had known, recovered, that nearly all died. Shortly afterwards, I accidentally learned that the practice of a physician who attended among these cases was, to give at first, fifteen grains of calomel at a dose. I did not marvel any more at the tale of mortality. But to come directly to the question, what is the remedy for this disease? I answer, emphatically, opium. This should be considered as the leading remedy in its treatment. The chief properties of opium are to relieve pain, produce somnolency, and cure inflammation of the mucous membranes; and I believe I may add, of most of the other tissues. But the most valuable, is that of curing inflammation and arresting inflammatory tendencies of the system. But, what! give opium before you have *cleansed* the stomach and bowels? I say yes, give it, it will cure them, filthy as they are. Give it, not in quarter grain doses, nor in half grain doses, but in from two to four grain doses, and repeat as often as necessary. Cure the primary disease, and the bile will flow beautifully again, and all will be right.

Thus, then, we have the remedy to answer the plain and unmistakeable indication of the disease, which used in time and in connection with cold water injections, blisters, the astringents, tannin, lead and zinc, creosote, &c., with the common effervescing soda powder to allay nausea and vomiting, where that exists, will rarely fail to secure a happy result. Try it, use it liberally, try it fairly, perseveringly, early in the attack, and the confidence of the community in the profession will be strengthened and increased by its success.

A good combination is made of two grains of opium and from half to one drop of creosote for a dose, to be repeated at proper intervals; and for children, an emulsion containing two drops of creosote to the ounce, with the addition of as much laudanum as may be desirable, to be given in tea-spoonful doses about three times a day.

